# Bulk Phase Proportion Governs the Wear Performance of PEO Coatings Grown on Biomedical Ti-6Al-4V Alloy

**DOI:** 10.3390/ma19183852

**Published:** 2026-09-10

**Authors:** José Roberto Ferreira Neto, Jhuliene Elen Muro Torrento, Carlos Eduardo da Silva, Fernanda de Freitas Quadros, Carlos Roberto Grandini, Sophia Alexandra Tsipas, Diego Rafael Nespeque Correa

**Affiliations:** 1 Laboratory of Anelasticity and Biomaterials, School of Sciences, São Paulo State University (UNESP), Campus Bauru, Bauru 17033-360, SP, Brazil; jose.roberto-ferreira@unesp.br (J.R.F.N.); jhuliene.torrento@unesp.br (J.E.M.T.); carlos.eduardo-silva2004@unesp.br (C.E.d.S.); ff.quadros@unesp.br (F.d.F.Q.); carlos.r.grandini@unesp.br (C.R.G.); 2 Department of Materials Science and Engineering and Chemical Engineering, University Carlos III of Madrid, IAAB, Avda. de la Universidad, 30, 28911 Leganés, Spain; stsipas@ing.uc3m.es

**Keywords:** biomaterial, Ti-6Al-4V, heat treatment, phase proportion, PEO, wear resistance

## Abstract

**Highlights:**

- Ti-6Al-4V alloy with distinct α and β proportions underwent PEO treatment.
- Wear tests indicated abrasive and adhesive wear mechanisms for untreated samples.
- PEO-treated samples depicted mainly an adhesive wear mechanism.
- Tailoring bulk phases and surface aspects by PEO can be beneficial for implants.

**Abstract:**

This study investigated the combined influence of the α/β phase proportion and Plasma Electrolytic Oxidation (PEO) on the wear behavior of Ti-6Al-4V alloy. Samples with distinct α/β phase ratios induced by previous heat treatments (600, 800, and 1000 °C) were subjected to dry sliding wear tests before and after PEO treatment. The untreated samples exhibited major abrasive and minor adhesive wear mechanisms, characterized by broad scratches and minor adhered debris. In contrast, PEO-treated samples exhibited predominant adhesive wear, indicating a significant tuning in the wear mechanism. The coefficient of friction (COF) was unaffected by phase proportions in untreated samples but was influenced by PEO treatment. Volume loss and wear rate were sensitive to phase composition, with the untreated samples heat-treated above 800 °C exhibiting the highest values, likely due to the retention of the metastable α′ phase. Conversely, PEO-treated samples demonstrated markedly reduced wear, confirming the protective role of the oxide layer. Energy-dispersive spectroscopy (EDS) revealed the counterbody’s particle adhesion in untreated tracks, whereas PEO-treated tracks retained the coating, evidencing superior wear resistance. These findings highlight that tailoring the α/β ratio through heat treatment combined with PEO treatment enhances wear performance, offering promising implications for clinical translation in biomedical implants.

## 1. Introduction

Due to their low elastic modulus, high biocompatibility, and excellent corrosion resistance, titanium (Ti) and its alloys are widely used as biomaterials [1]. Among these, the Ti-6Al-4V alloy (wt%) [2], also known as Ti grade 5, is the most widely used worldwide. It comprises α- and β-phases and is frequently used in orthopedic implants. Nevertheless, a relevant concern is the possible release of toxic aluminum (Al) and vanadium (V) ions from long-term implants [3]. This highlights the importance of research aimed at mitigating the alloy’s corrosion and wear in the human body.

Wear resistance is vital to the durability and performance of coated surfaces, particularly in applications involving friction or repeated contact, such as biomedical implants [4]. In this sense, the wear response of coated biomedical implants is influenced by various factors, including hardness, adhesion, and thickness, which collectively determine the lifespan of the implantable material [5,6]. In this scenario, surface treatments can significantly affect wear resistance, thereby determining the implant’s effectiveness under challenging human body conditions and warranting further investigation in current research.

Among the many surface treatment techniques available for Ti and its alloys, plasma electrolytic oxidation (PEO) is an effective method for tuning the surface properties [7]. The process is based on the application of a high anodic voltage to the surface in a conductive electrolyte. Due to dielectric breakdown of the anodic oxide coating, a thick, porous, and well-adhered TiO_2_ layer is formed on the surface [8,9]. Moreover, PEO enables the incorporation of elements such as calcium (Ca) and phosphorus (P) into the surface, thereby enhancing biocompatibility and bioactivity [10,11]. Although the benefits of PEO treatment in enhancing the corrosion and tribological properties of Ti alloys in simulated body fluids are well documented [12,13,14], the influence of the α and β phases on the growth mechanisms of the oxide layer and the corresponding effects on surface characteristics has not been investigated in depth. 

In our previous study [15], we subjected Ti-6Al-4V alloy to heat treatments to maintain distinct α- and β-phase fractions, followed by PEO treatment in a Ca- and P-rich electrolyte. The corresponding PEO-treated samples were sensitive to the bulk’s phase proportion, particularly with respect to surface chemical composition, crystallinity, roughness, wettability, and surface energy. Promising results were also observed in corrosion tests conducted in a physiological solution (0.9% NaCl), indicating that combining bulk and surface processing can confer advantages for biomedical Ti implants. From this point, the current study aims to further investigate the effects of PEO treatment and the bulk-phase proportion of Ti-6Al-4V alloy on wear behavior and to confirm their applicability in biomedical applications.

## 2. Materials and Methods

### 2.1. Sample Processing

Square-shaped samples measuring 20 mm × 20 mm × 3 mm, made from Ti-6Al-4V alloy (Sandinox Ltd., Sorocaba, Brazil), served as substrates. The samples were polished with SiC papers (3M Science Inc., Sumaré, SP, Brazil) ranging from #220 to #1200 mesh, then ultrasonically cleaned in acetone for 5 min, and air-dried. Some samples remained in their as-received state, whereas others underwent an initial heat treatment at 1000 °C for 12 h to promote microstructural homogenization, followed by slow furnace cooling. Subsequently, the samples were divided into three groups, each heat-treated at 600, 800, or 1000 °C for 30 min, and then rapidly cooled in water. All heat treatments were done under a vacuum of 10^−6^ Torr. The PEO treatment was conducted in a stainless-steel tank (cathode) equipped with a refrigeration system, with the samples connected to the positive terminal (anode). The surface treatment was conducted using an AC power source (MAO-30, Plasma Technology Ltd., Hong Kong, China) set to pulsed mode (+480 V/0 V), 100 Hz, with a 60% duty cycle for 120 s. The electrolyte contained 0.2 mol·L^−1^ calcium acetate (Sigma-Aldrich Inc., USA) and 0.02 mol·L^−1^ sodium glycerophosphate (Sigma-Aldrich Inc., Saint Louis, MO, USA) in deionized water. After treatment, the samples were rinsed with distilled water and dried in a hot-air oven.

### 2.2. Wear Testing

Wear resistance was assessed through dry sliding wear tests performed on a ball-on-plate tribometer (UMT, Bruker Corp., Billerica, MA, USA) with linear reciprocating motion, following the ASTM G99-17 standard [16]. The samples (*n* = 2/group) acted as the moving body, while the static body served as the counterbody. Tests were conducted under unlubricated conditions in ambient air. The wear resistance measurement involved rubbing a 5 mm alumina (Al_2_O_3_) ball under a 2 N load at 1 Hz for 1800 s, covering a total stroke length of 10 mm. Optical (BX51M, Olympus Corp., Center Valley, PA, USA), confocal (DM3D model, Leica Corp., Teaneck, NJ, USA), and scanning electron microscopies (SEM, EVO LS30, Carl Zeiss Inc., Dublin, CA, USA) were used to analyze the wear marks (*n* = 10/group), with some chemical details investigated by X-ray energy-dispersive spectroscopy (EDS), using a detector (Bruker Corp., Billerica, MA, USA) coupled with SEM equipment (XL-30 FEG, Philips Corp., Cambridge, MA, USA). Then, the volume loss was subsequently estimated according to the procedures described by Urena et al. [17].

In brief, to calculate the volume loss, the area loss for each 2D profile was obtained from Equation (1), where ***A_w_*** is the area loss for each 2D profile (mm^2^), ***Y*** is the depth (mm), ***n*** is the number of measurements, and ***X*** is the width (mm).

(1) $$A_{w}=\sum_{i=1}^{n}0.5\cdot \left(Y_{i}-Y_{i-1}\right)\cdot (X_{i}-X_{i-1})$$

Then, the average depth value ($\bar{D}$) was calculated based on Equation (2), where $\bar{W}$ is the average width of each wear track measured in the 2D profiles, and ($\bar{A_{W}}$) is the average area loss of three 2D profiles for each wear track.

(2) $$\bar{D}=\bar{A_{w}}/\bar{W}$$

The volume loss was estimated with Equation (3), where ∆***V*** is the total volume loss for each wear track (mm^3^); ***l*** is the total stroke length, which was constant for all tests (10 mm); and ***R*** is the radius of the ball.

(3) $$∆V=\left[\left(1/3\right)\cdot \pi \cdot {\bar{D}}^{2}\cdot \left(3R-\bar{D}\right)\right]+\bar{A_{w}}\cdot l$$

The specific wear rate was calculated with Equation (4), where ***W_S_*** is the specific wear rate (mm^3^/Nm); ***P*** is the normal load, which was 2 N for all tests; and ***S*** is the distance traveled, 10 mm.

(4) $$W_{s}=∆V/P\cdot S$$

## 3. Results and Discussion

**Figure 1:**
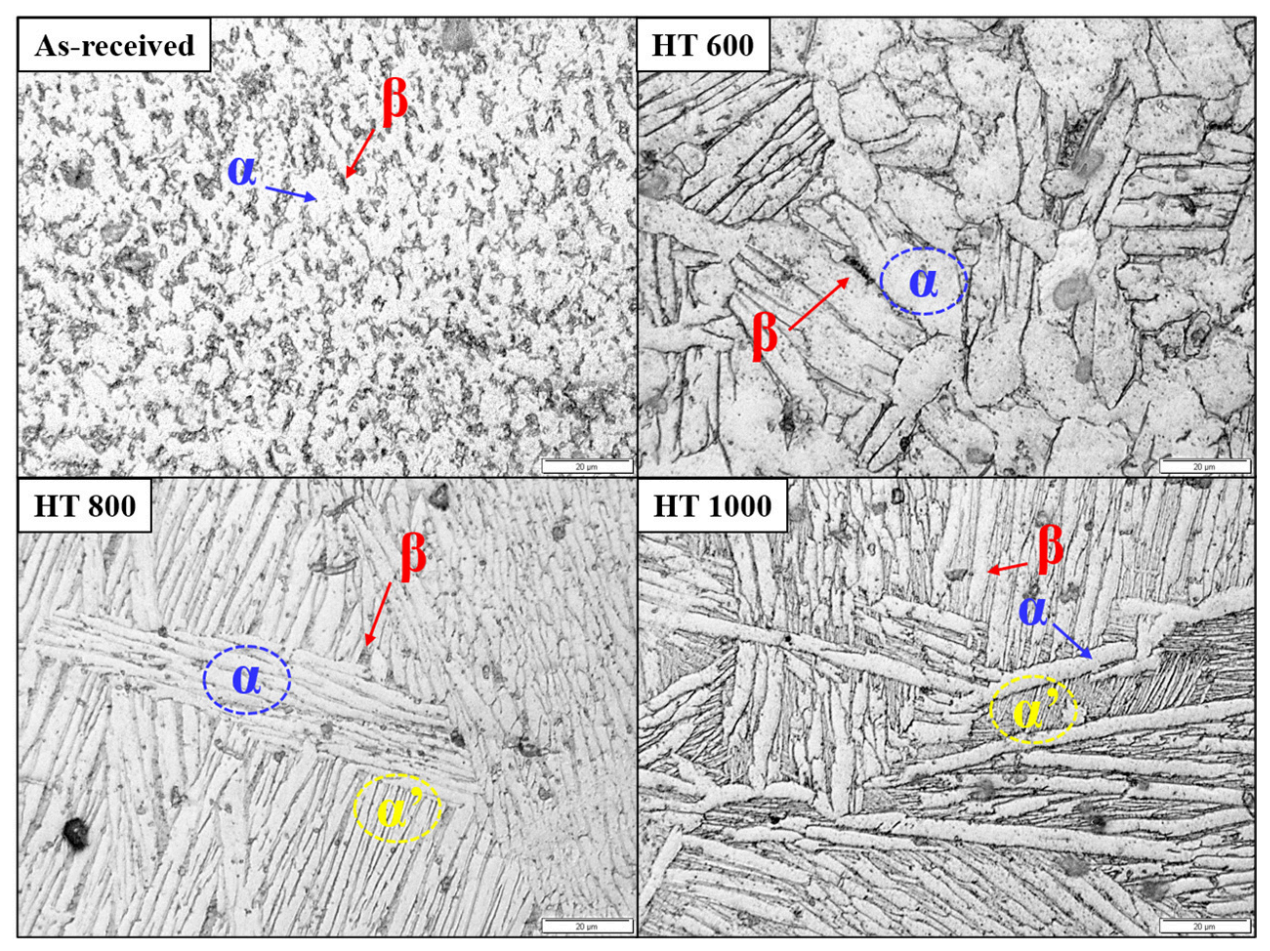
Microstructural analysis of the untreated samples. Color labels and arrows indicate the distinct Ti’s phases.

Table 1 displays the samples’ nomenclature and some microstructural and topographical features reported in our previous study [15], while Figure 1 shows the optical micrograph of each untreated sample, which agrees well with the predicted microstructure of the Ti-6Al-4V alloy submitted to solution treatments [18]. The microstructural analysis was done after conventional metallographic procedures involving SiC sandpaper grinding, alumina colloidal suspension polishing, and etching in Kroll’s acid solution.

**Figure 2:**
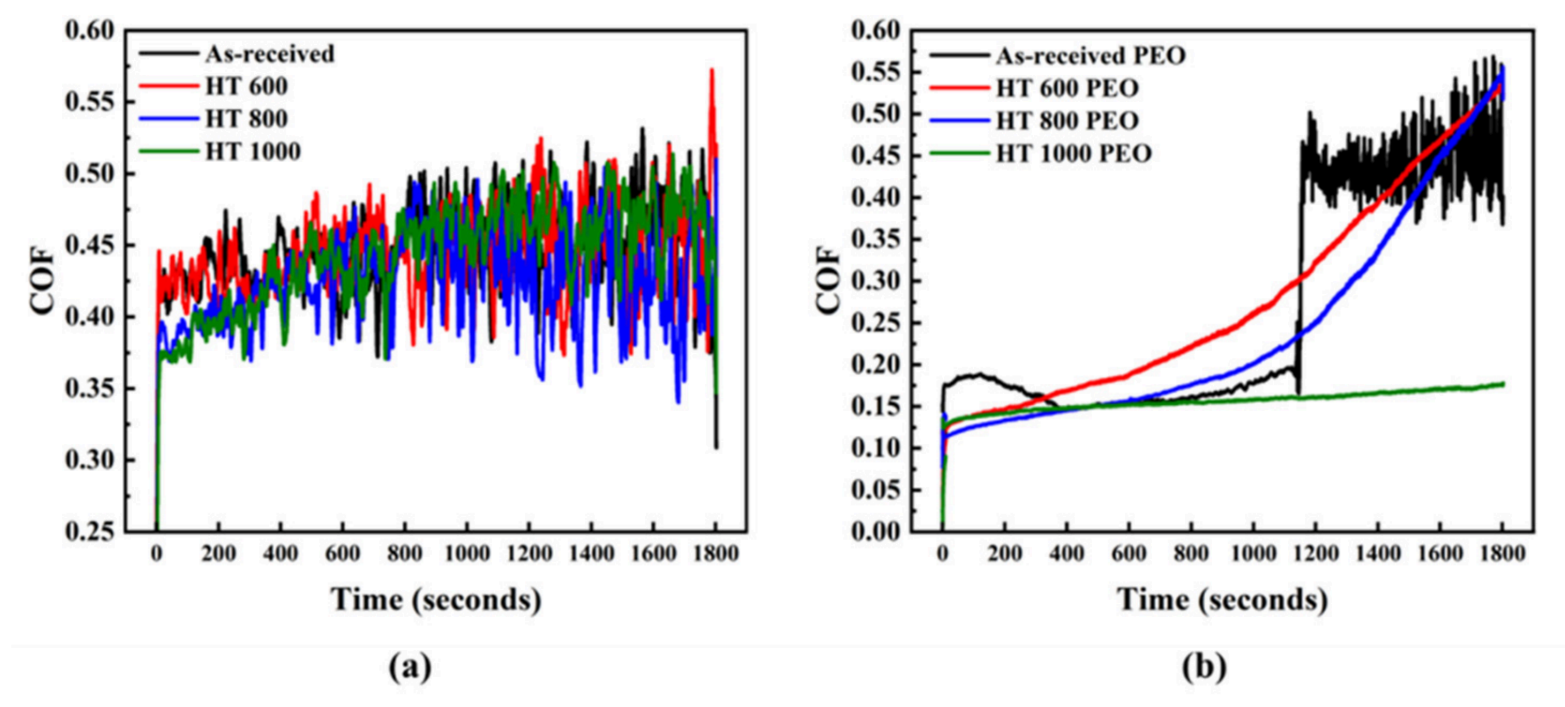
Comparison of the representative COF values for the untreated (**a**) and PEO-treated (**b**) samples.

The coefficient of friction (COF) was monitored during the wear tests, and the curves for each sample are shown in Figure 2. Samples without PEO exhibited similar COF values, ranging from 0.35 to 0.55, consistent with the typical behavior of metallic surfaces. This suggests that the initial phase proportion did not significantly affect the COF under this load condition. In contrast, PEO-treated samples differ significantly across conditions. The as-received sample initially had a COF of approximately 0.15, lower than that of untreated metals, indicating a coating’s protective effect and enhanced wear resistance. At approximately 1200 s, the COF values increased sharply to 0.40–0.50 due to rupture of the oxide layer by the counterbody. The heat-treated sample had lower COF values than the as-received PEO-treated one. Those treated at 600 °C and 800 °C exhibited a gradual increase in COF, indicating that the oxide layer was not fully removed and continued to protect the metal, likely due to improved adhesion to the substrate. The sample treated at 1000 °C maintained a stable COF throughout the test, possibly indicating optimal protective effect and wear resistance. According to Qin et al. [19], PEO treatment has been shown to enhance the friction and wear characteristics of Ti-6Al-4V alloy, with the most effective strategies involving the control of electrical parameters (voltage, current, and time) and electrolyte parameters (additives and concentration). However, the obtained results indicate that the bulk phase composition significantly affects the performance of the PEO coating. When the bulk phase is deformed, with α matrix + β precipitates, the PEO coating fails after 1200 s, whereas when the bulk phase consists of recrystallized α plates + β precipitates or refined α plates + coarse β precipitates, the PEO coating exhibits gradual degradation. On the other hand, when the bulk is composed of thin α plates + α’ needles + coarse β precipitates, the PEO seems to withstand the wear. It can therefore be concluded that the adhesion and/or growth of the PEO coating is affected by the underlying phase composition and microstructure. Further nanoindentation tests could be an interesting tool for correlating the coating’s mechanical properties with its tribological behavior.

**Figure 3:**
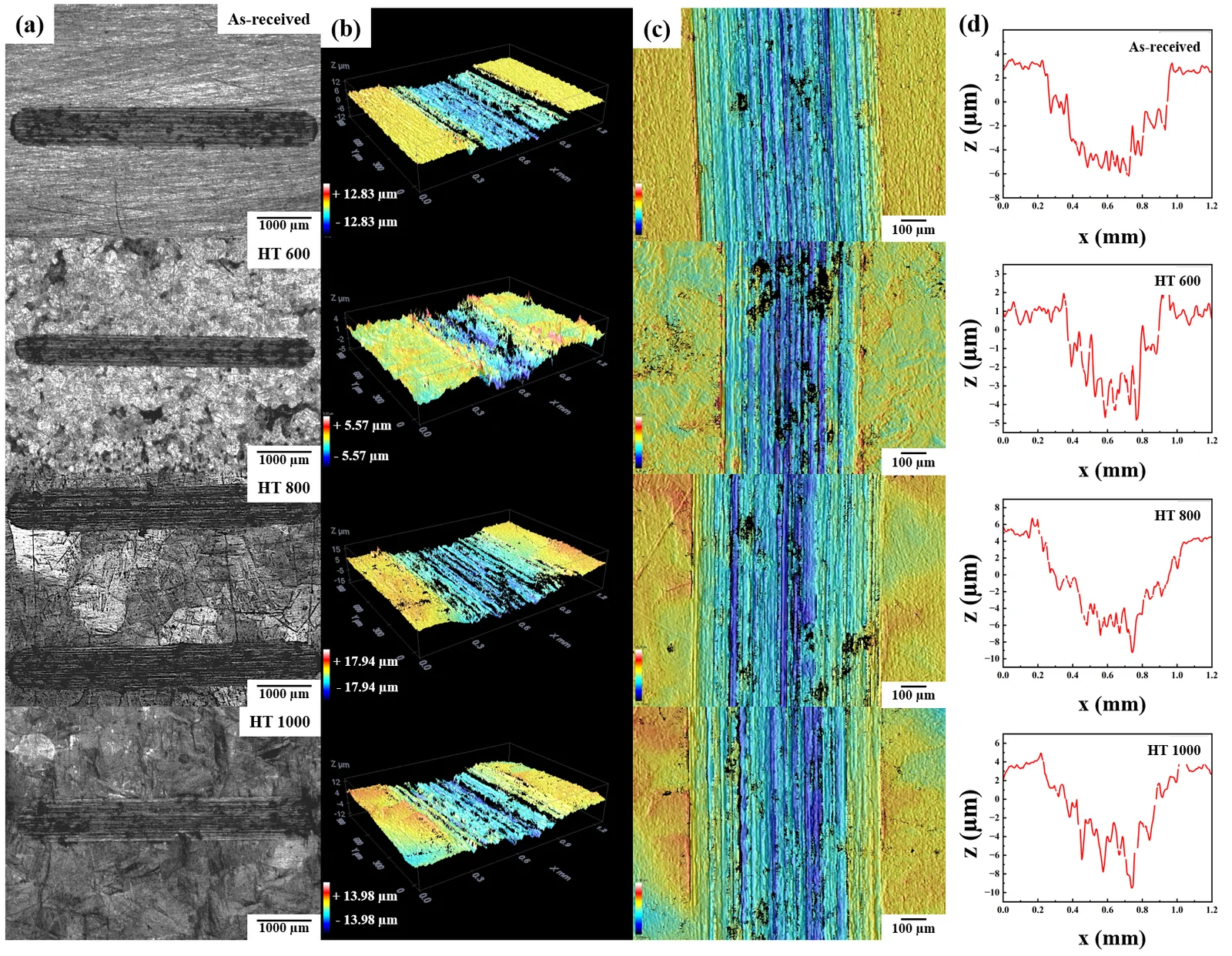
Topographical aspects of the wear track for the untreated samples: optical image of wear track (**a**), 3D optical microscopy indicating the wear track depth (**b**), 2D microscopy of wear track (**c**), and wear track cross-sectional depth (**d**).

Figure 3a presents optical microscopy images that clearly show how the counterbody affected the untreated samples, producing a track approximately 1 mm wide. Although the COF values stayed similar, slight variations in track size are visible. The 3D and 2D confocal images (Figure 3b,c) depict scratches and adhered layers along the track, resulting in an uneven surface. The images also demonstrate differences in the depth and width of scratches and layers, reflecting a significant contribution to the existing phases. The Z-profile curves (Figure 3d) exhibit an irregular topography, with a predominantly U-shaped profile typical of this type of wear testing. Scratches are associated with abrasion, whereas adhered layers indicate an adhesive wear mechanism, which is typical of Ti and its alloys submitted to wear testing [20]. Xu et al. [21] found that abrasive wear was the main mechanism in additive-manufactured Ti-6Al-4V alloy at room temperature, transitioning to adhesion and oxidative mechanisms under rising temperatures, agreeing well with our current study.

**Figure 4:**
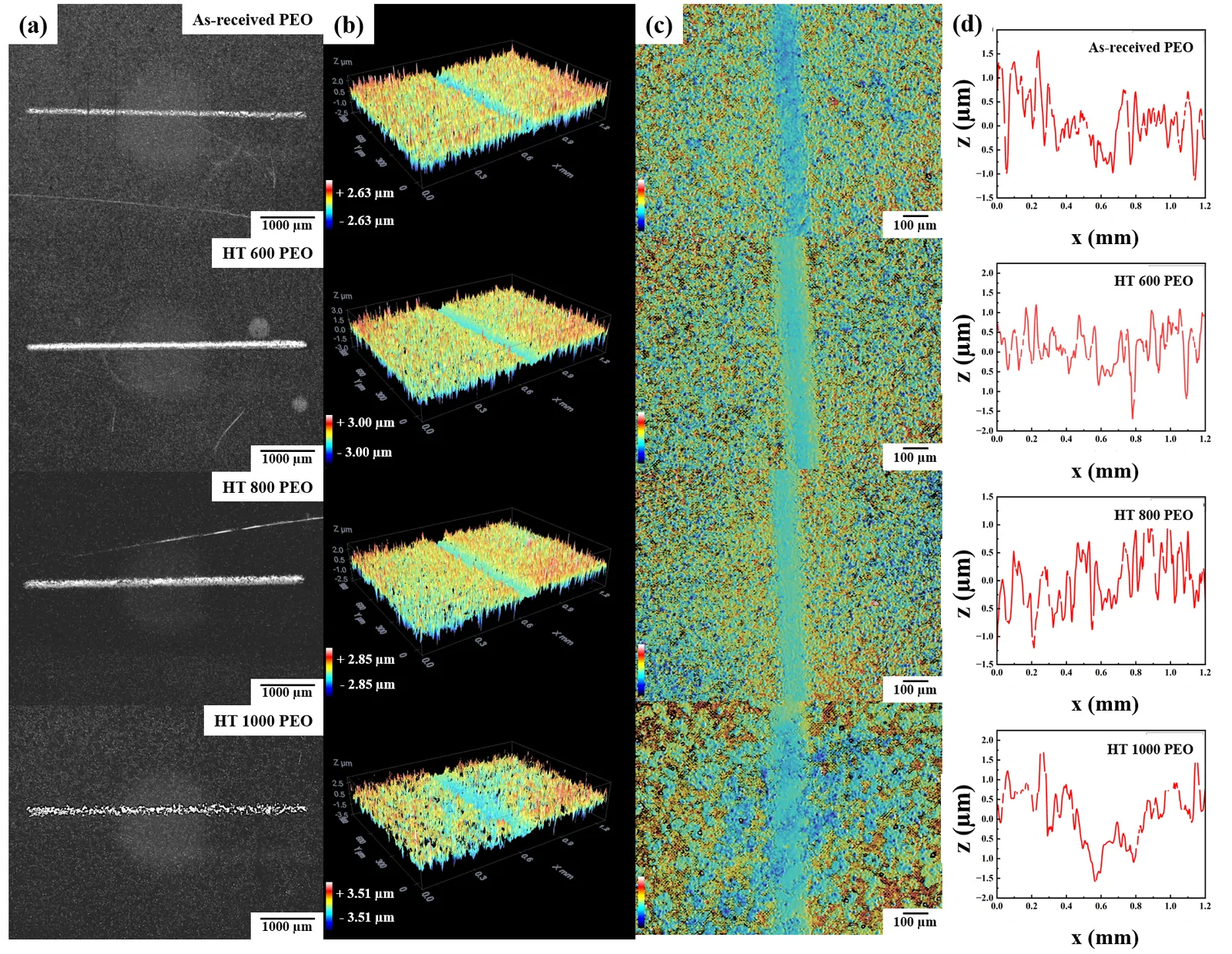
Topographical aspects of the wear track for the PEO-treated samples: optical image of wear track (**a**), 3D optical microscopy indicating the wear track depth (**b**), 2D microscopy of wear track (**c**), and wear track cross-sectional depth (**d**).

Optical images of the PEO samples (Figure 4a) showed wear tracks significantly narrower than those in untreated samples, with no delamination signal, indicating enhanced wear resistance of the PEO coatings. The 3D and 2D confocal images (Figure 4b,c) reveal a rough surface with a wear track approximately 100 µm wide. No scratches were clearly observed in the inner portion of the wear track, indicating a minor abrasive wear mechanism. The Z-profile (Figure 4d) indicates that the wear track is shallow, with a smooth counterbody mark observed after the test. The results indicated that adhesive wear was the predominant wear mechanism in the PEO-treated samples, consistent with the findings of Asl et al. [22], who investigated the wear mechanisms of oxidized commercially pure Ti (CP-Ti), Ti-6Al-4V, and Ti-45Nb alloys. The authors found that the higher hardness of the TiO_2_ layer resulted in superior resistance to surface degradation and attenuation of third-body effects on the adhesion and abrasion wear mechanisms. Similar results were found by Santos et al. [23], who evaluated the wear resistance of PEO-treated and additive-manufactured Ti-6Al-4V alloy under wear tests in a ball-on-plate configuration. The authors found the same adhesive mechanism in the coatings, with a sensible effect of the α’ phase retained by the L-PBF (laser powder bed fusion) process.

**Figure 5:**
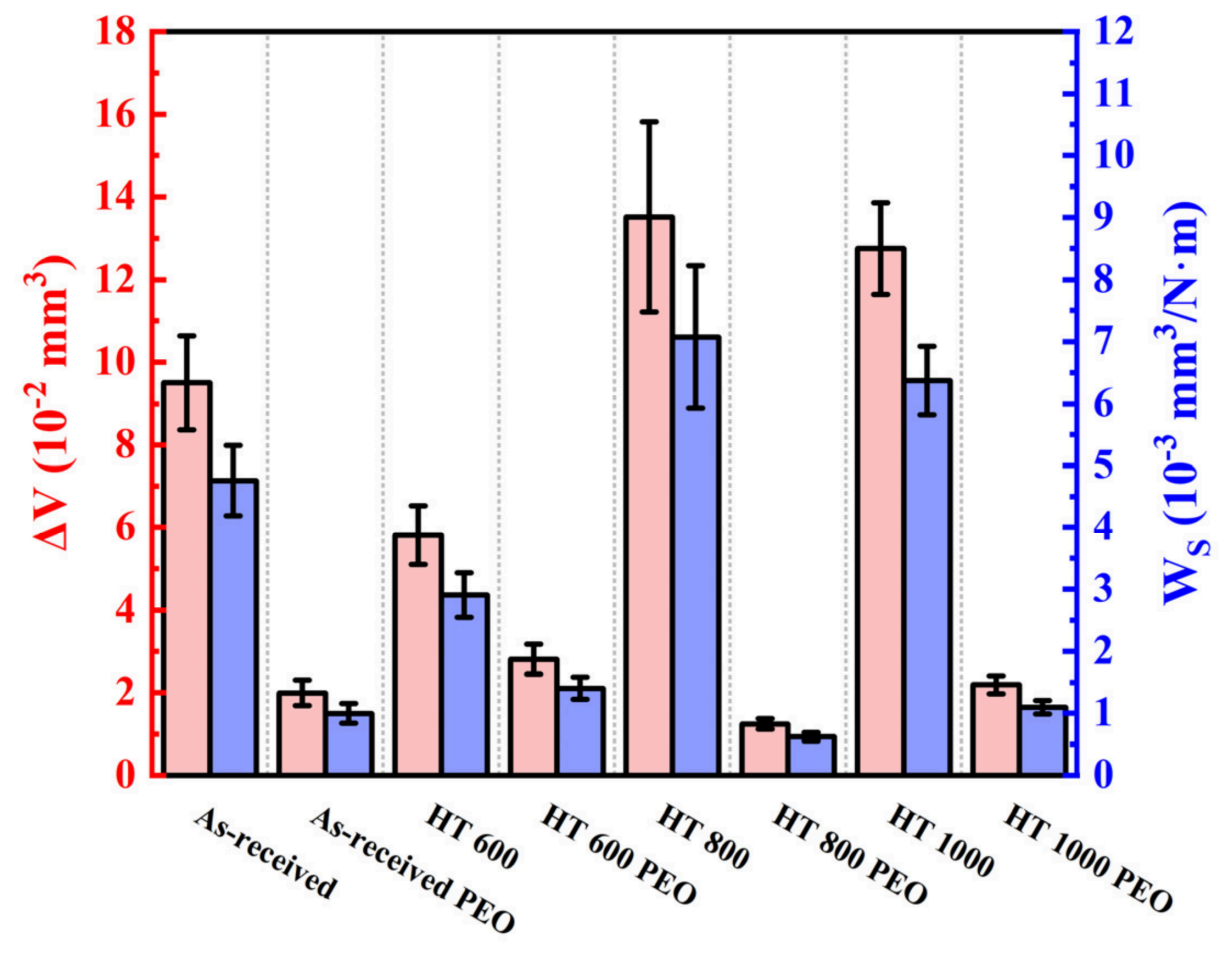
Calculated volume loss and wear rate of the samples.

The corresponding volume loss and wear rate, estimated from the wear track geometry, are compared in Figure 5. The superior wear resistance of the PEO-treated samples relative to the untreated ones is evident. However, small differences in volume loss and wear rate with respect to the processing history are also observed. The as-received sample exhibited intermediate values, likely due to residual stress generated by thermomechanical processing, which can induce hardening mechanisms in the material, as demonstrated by Rai et al. [24]. The sample treated at 600 °C (majority α phase) exhibited the lowest volume loss and wear rate, whereas the samples treated at 800 °C and 1000 °C (α + α’ + β phase) showed the highest values. This augmentation can be linked to the possible existence of a metastable α’ phase, which can be retained under fast cooling from high temperatures [25] and possesses a hard nature compared to the α and β phases [26]. The PEO samples exhibited a significant decrease in volume loss and wear rate, consistent with the greater mechanical strength of the oxide layer, which was primarily composed of TiO_2_. The 600 °C sample treated with PEO showed a slight increase relative to the as-received PEO sample, while those heat-treated at 800 °C and 1000 °C and PEO-treated possessed almost the same values. Although further tests are required to elucidate the effect of the bulk phase proportion on the wear resistance of PEO coatings, it can be inferred that the PEO treatment resulted in clear benefits in volume loss and wear rate for the Ti-6Al-4V alloy, which are useful for biomedical applications.

**Figure 6:**
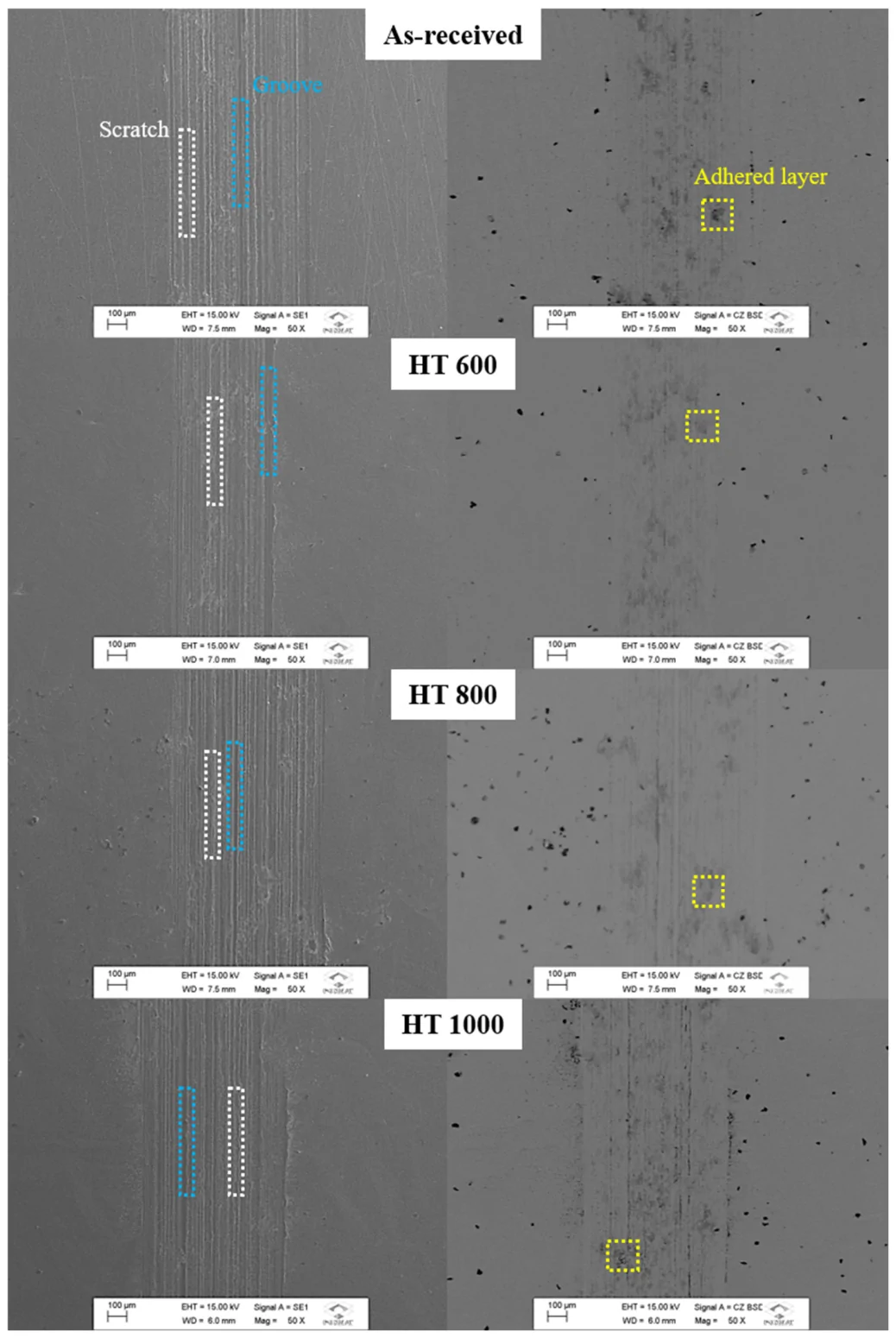
SEM imaging of the wear tracks for the untreated samples: secondary electron (SE) in the left and backscattered electron (BSE) beam modes in the right. The color boxes indicate distinct marks along the wear track.

A zoomed view of the wear tracks in the untreated samples is shown in Figure 6, with SEM images acquired in the SE and BSE modes. The wear tracks in the SE images are similar, exhibiting grooves and scratches oriented toward the counterbody’s motion, confirming the abrasive wear mechanism. Furthermore, some adhered particles are observed along the wear tracks in BSE images, appearing dark due to Z-contrast from electron-beam scattering. These adhered particles can be oxide particles originating from the counterbody (Al_2_O_3_) and metallic debris removed from the metal that oxidized at high temperatures and were then redeposited on the surface. The induced oxidation of metallic debris is consistent with the study by Li et al. [27], who investigated the formation mechanisms of debris in Ti-6Al-4V alloy during cutting.

**Figure 7:**
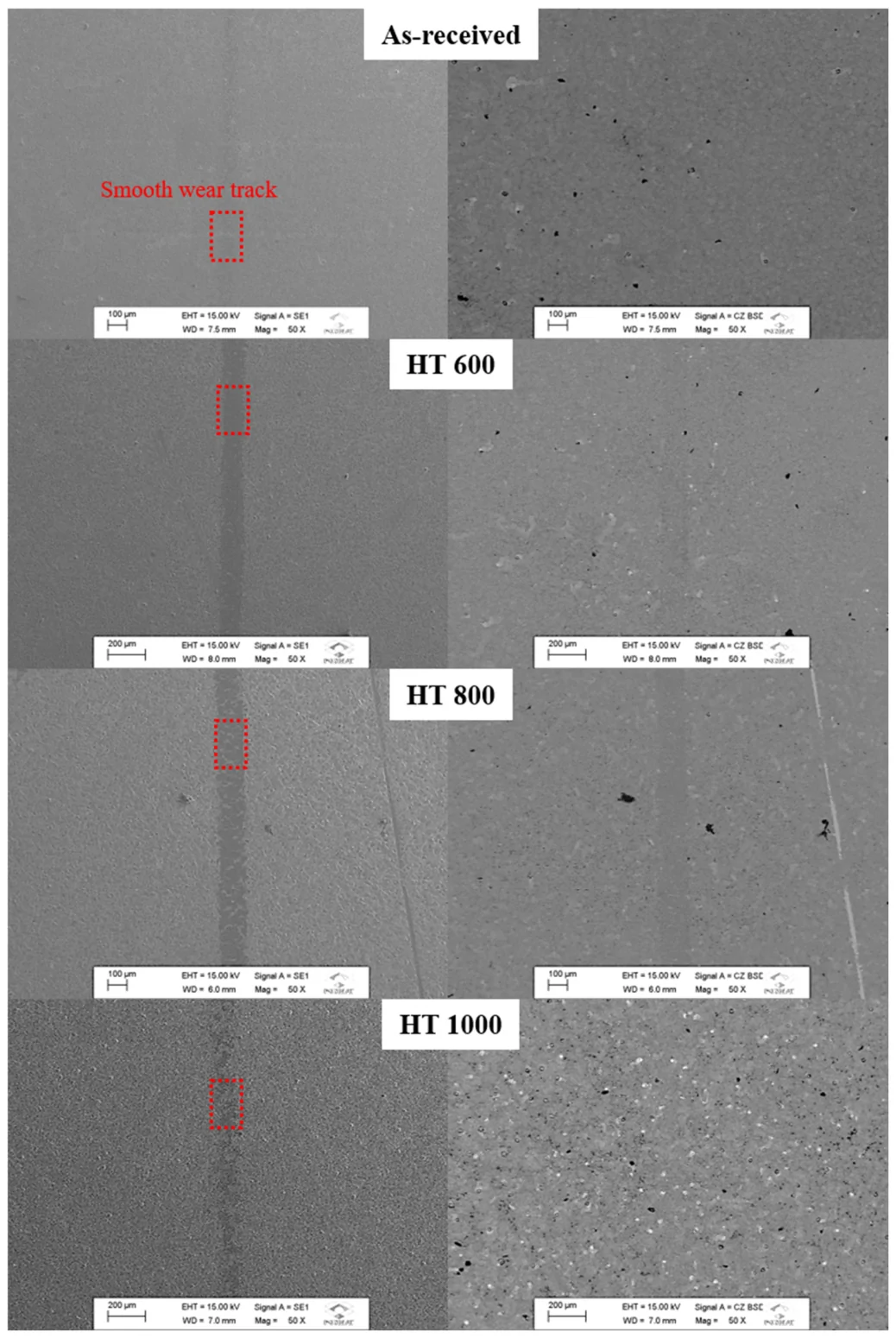
SEM imaging of the wear tracks for the PEO-treated samples: secondary electron (SE) in the left and backscattered electron (BSE) beam modes in the right. The color boxes indicate distinct marks along the wear track.

In the same way, further details of the wear tracks in the PEO-treated samples are shown in Figure 7. Overall, the wear tracks appeared smooth and shallow compared to the untreated samples. Instead of scratches or grooves, only adhered marks (typical of adhesive wear) are clearly observed in the wear tracks, suggesting reduced abrasive wear and a predominance of the adhesive wear mechanism. Furthermore, the BSE images show a smooth Z-contrast signal from adhered particles, confirming the presence of adhesion wear. As reported by Qin et al. [19] and confirmed in our previous studies [28], beneath the porous outer layer, the PEO coating contains a dense oxide layer that contributes to surface protection under wear and frictional conditions, thereby enhancing wear resistance.

**Figure 8:**
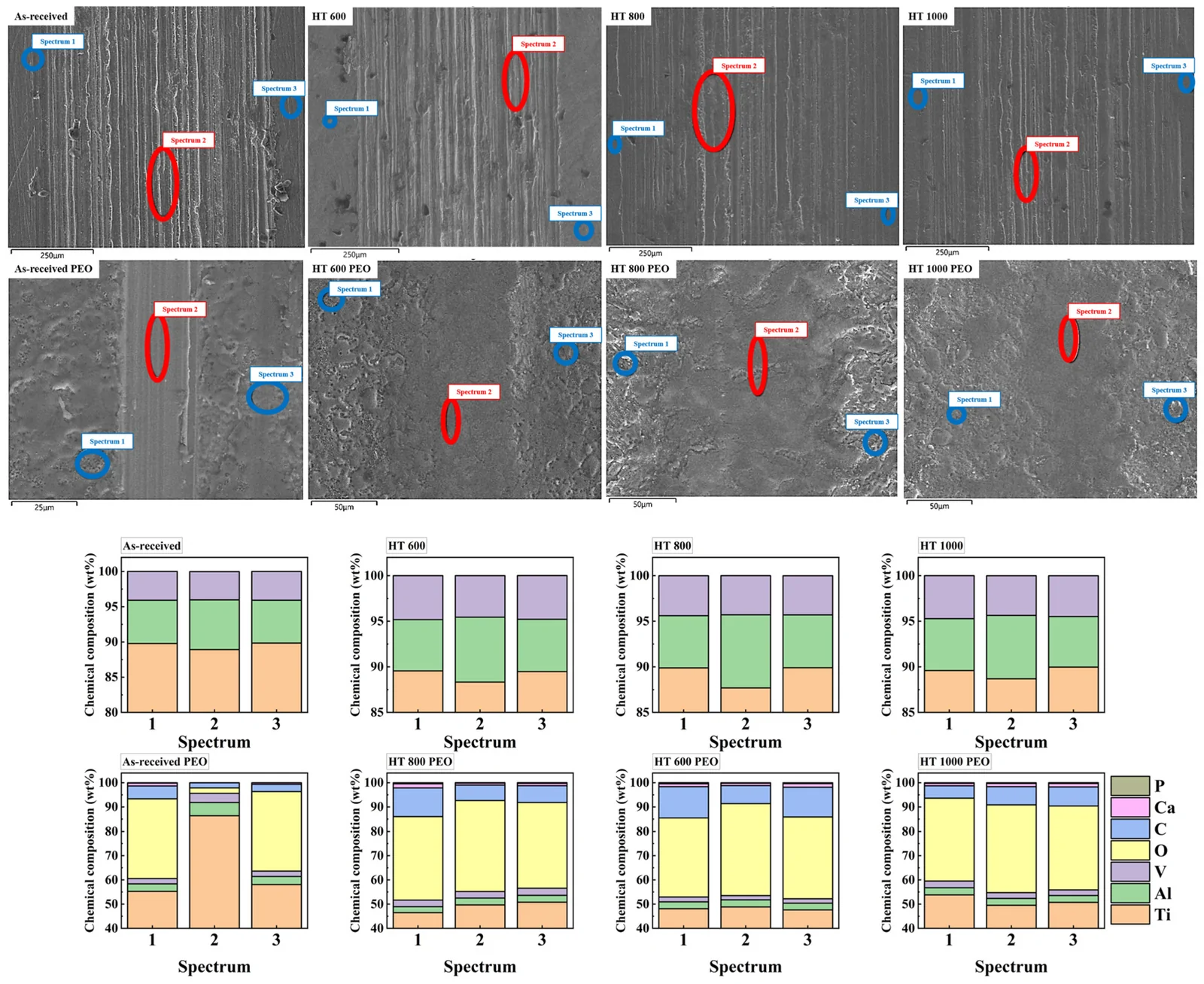
Semi-quantitative EDS analysis of the wear tracks.

Chemical details of the wear tracks are shown in Figure 8, where punctual EDS analyses were used to determine chemical compositions semi-quantitatively. The corresponding values can be checked in the Supplementary Materials (Table S1). Spectra 1 and 3 were taken outside the wear track, while spectrum 2 was taken within. For the untreated samples, the proportions between the alloying elements (Ti, Al, and V) remained closer to the nominal values for the Ti-6Al-4V alloy (wt%). However, spectrum 2 showed an increase in Al relative to Ti, likely indicating the presence of Al particles removed from the counterbody. This fact is confirmed by the image of the worn surface on the alumina ball after wear testing (see Figure S1 in the Supplementary Materials). In contrast, for the PEO-treated samples, spectra 1 and 3 showed high O and Ti and a depletion of Al and V, indicating the presence of Ti oxides (primarily TiO_2_) on the surface. The presence of minor amounts of C, Ca, and P supports the formation of calcite and phosphate, as reported in our earlier study [15]. However, except for the as-received PEO samples, the other samples retained nearly the same chemical composition as in spectrum 2, indicating that the counterbody movement did not entirely remove the PEO coating and expose the metallic surface, thereby ensuring proper adhesion of the coating to the substrate and wear resistance. The good adhesion of the coatings was also confirmed by ultrasonic bath testing, in which the surface remained unchanged after 30 min of immersion (see Figure S2 in the Supplementary Materials). Residual stress in the as-received PEO sample can adversely affect the coating’s adhesion, leading to significant coating removal induced by the counterbody and to an increase in the Ti chemical proportion in spectrum 2. Lastly, EDS analysis confirmed the superior wear resistance of the PEO-treated samples, regardless of the bulk-phase proportion. Similar results were obtained by Sousa et al. [29], who evaluated the effects of Ca and P in the PEO coating of CP-Ti on the tribocorrosion behavior. The authors found that the presence of O atoms (as oxides) indicates that PEO coatings provide a protective effect on the surface, thereby ensuring superior performance in biomedical implants. 

**Figure 9:**
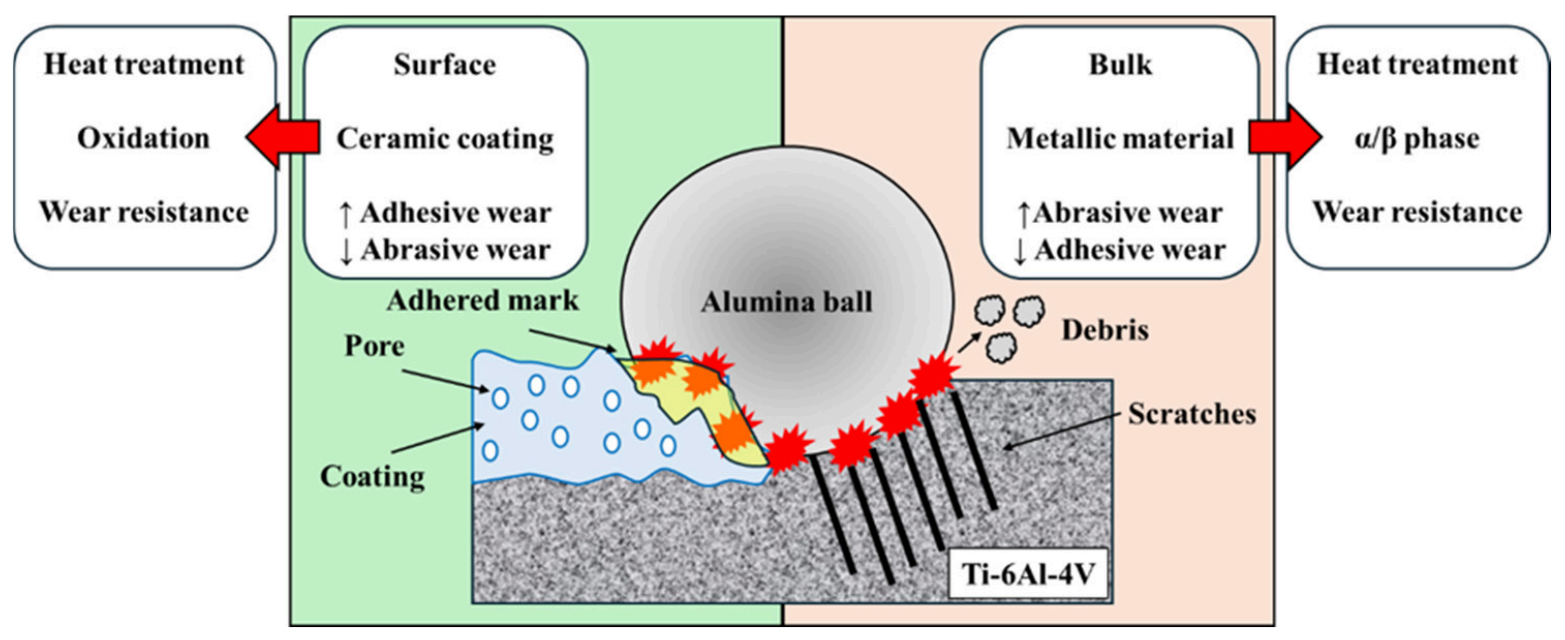
Schematic diagram of the wear mechanism evolved in the bulk and surface.

The overall results are summarized in the schematic diagram shown in Figure 9, which are in line with the report of Rosenkranz et al. [30], who detailed how the physics and chemistry of the coating/substrate interface can play a primary role in the tribological aspects of the material. The untreated samples exhibited mainly grooves and scratches from abrasive wear, with minor layers attributable to adhesive wear. The α/β phase proportion induced by the heat treatment slightly affected wear resistance, as these phases possess distinct hardness and elastic modulus, thereby mitigating surface damage from the alumina ball. On the other hand, the PEO-treated samples exhibited a pronounced enhancement in wear resistance due to the protective effect of the anodic oxide layer. Adhesive wear was the main mechanism, directly dependent on the α/β phase proportion, as these phases can alter the oxidation process and thereby influence the coating/substrate interface. Therefore, controlling bulk phases in Ti-6Al-4V alloy through heat treatment can be a smart strategy to tune the wear resistance of PEO coatings, thereby mitigating debris and ion release from the implant into human blood and avoiding adverse allergic and toxic reactions. Further tribocorrosion tests are welcome to evaluate the response of these materials under synergistic corrosion and wear conditions.

## 4. Conclusions

In this study, Ti-6Al-4V samples with distinct α- and β-phase proportions were subjected to PEO in a Ca- and P-rich electrolyte and subsequently underwent a dry sliding wear test. From the results obtained in this study, it is possible to summarize the following:

- The bulk phase proportions did not affect the COF values of the untreated samples but did affect the PEO-treated ones, playing a role in the adherence at the interface with the coating.
- The wear track of the untreated samples depicted scratches and adhered debris resulting from the abrasive and adhesive wear mechanisms, respectively. In contrast, the PEO-treated samples exhibited only deformed marks, indicating predominantly adhesive wear despite the abrasive wear mechanism. Therefore, the heat treatment improved the coatings’ integrity, as no debris, abrasion, or fractures were observed in the tracks.
- The volume loss and wear rate were sensitive to the bulk phase proportion, with the untreated HT 800 and HT 1000 samples possessing large values, probably due to the retention of the metastable α’ phase, which is predicted by the pseudo-binary phase diagram and is well known for its hardening effect compared to the α and β phases. In contrast, the PEO-treated samples exhibited significantly lower values, indicating that the oxide layer successfully protects against wear.
- The punctual EDS chemical analyses evidenced the presence of adhered particles from the counterbody in the wear track region of the untreated samples. For the PEO-treated samples, the presence of an oxide layer in the wear track after the test confirmed the coating’s good adhesion to the substrate and superior wear resistance.
- The results show that the α and β phase ratios, generated by the heat treatment, combined with the PEO treatment, resulted in beneficial effects on the wear resistance, which may help improve the quality of biomedical implants produced with the commercial Ti-6Al-4V alloy. Further studies on the chemical composition and hardness of the coatings, and their correlations with tribocorrosion and biological aspects, are recommended to provide a clearer view of their applicability in the field.

## Figures and Tables

**Table 1:** Labeling and details of the samples after heat treatment (HT) and surface treatment (PEO). Further details can be found in our earlier study [15].

Samples	Condition	Description
As-received	Raw metal	Deformed α matrix + β precipitates
HT 600	Heat-treated at 600 °C	Recrystallized α plates + β precipitates
HT 800	Heat-treated at 800 °C	Refined α plates + β precipitates
HT 1000	Heat-treated at 1000 °C	Thin α plates + α’ needles + β precipitates
As-received PEO	PEO-treated raw metal	Porous TiO_2_ layer, thickness of 8–9 μm, with equal amounts of anatase and rutile, and Ca- and P-rich
HT 600 PEO	Heat-treated at 600 °C and PEO-treated	
HT 800 PEO	Heat-treated at 800 °C and PEO-treated	
HT 1000 PEO	Heat-treated at 1000 °C and PEO-treated	

## Data Availability

The original contributions presented in this study are included in the article/Supplementary Materials. Further inquiries can be directed to the corresponding author.

## Supplementary Materials

The following supporting information can be downloaded at: https://www.mdpi.com/article/10.3390/ma19183852/s1, Figure S1: Representative SEM images of the worn surface of the alumina ball after the wear testing of the samples. Images were taken in the SE (left) and BSE (right) modes, with insets showing a zoomed view of the worn surface. The worn region was similar to all samples and confirms the degradation of the alumina ball during the wear tests. Figure S2: Topographical features of the samples before and after ultrasonic bath in distilled water for 30 min. The large images were obtained by SE-SEM, while the insets were acquired with a conventional camera. The similarity between the groups demonstrates the high adhesion of the coatings to the substrate. Table S1: Chemical proportion values of the samples acquired by punctual EDS (resolution of 0.01 at%) in the worn surface (region 2) and the lateral undamaged surfaces (regions 1 and 2). The values were used in the semi-quantitative analysis of the wear track (Figure 8).

## Abbreviations

The following abbreviations are used in this manuscript:

PEO	Plasma electrolytic oxidation
COF	Coefficient of friction
EDS	Energy-dispersive spectroscopy
SEM	Scanning electron microscopy
