# Supplementary material for: Bulk Phase Proportion Governs the Wear Performance of PEO Coatings Grown on Biomedical Ti-6Al-4V Alloy
Source: Materials (Basel). 2026 Sep 10;19(18):3852. doi: 10.3390/ma19183852 (PMC13608053; doi:10.3390/ma19183852)
Supplement: Supplementary file 1 [file materials-19-03852-s001.zip › materials-4506053-supplementary.pdf]

Supplementary Material

Table S1 – Chemical proportion values of the samples acquired by punctual EDS (resolution of 0.01 at%) in the worn surface (region 2) and the lateral undamaged surfaces (regions 1 and 2). The values were used in the semi-quantitative analysis of the wear track (Fig. 8).

Sample	Spectrum	Chemical proportion (at%)						
		Ti	Al	V	O	C	Ca	P
	1	89.78	6.15	4.08	-	-	-	-
**As-received**	2	88.92	7.06	4.06	-	-	-	-
	3	89.85	6.09	4.06	-	-	-	-
	1	55.27	3.11	2.21	32.78	5.30	1.16	0.18
**As-received PEO**	2	86.38	5.44	3.78	2.28	2.10	0.03	0.01
	3	58.09	3.33	2.21	32.71	3.01	0.57	0.10
	1	89.56	5.62	4.83	-	-	-	-
**HT 600**	2	88.33	7.12	4.56	-	-	-	-
	3	89.48	5.79	4.79	-	-	-	-
	1	48.12	2.84	2.00	32.50	12.89	1.12	0.47
**HT 600 PEO**	2	48.86	2.94	1.72	37.84	7.43	1.01	0.22
	3	47.62	2.78	1.81	33.63	12.29	1.51	0.37
	1	89.88	5.73	4.39	-	-	-	-
**HT 800**	2	87.69	8.02	4.29	-	-	-	-
	3	89.90	5.80	4.30	-	-	-	-
	1	46.49	2.46	2.72	34.34	11.87	1.68	0.46
**HT 800 PEO**	2	49.70	2.81	2.73	37.41	6.36	0.82	0.19
	3	50.80	2.84	2.94	35.27	6.94	0.97	0.27
	1	89.60	5.69	4.72	-	-	-	-
**HT 1000**	2	88.69	6.95	4.36	-	-	-	-
	3	89.97	5.55	4.48	-	-	-	-
	1	53.82	2.98	2.74	34.07	5.11	1.04	0.25
**HT 1000 PEO**	2	49.55	2.83	2.38	36.10	7.50	1.35	0.30
	3	50.73	2.82	2.34	34.51	7.86	1.40	0.36

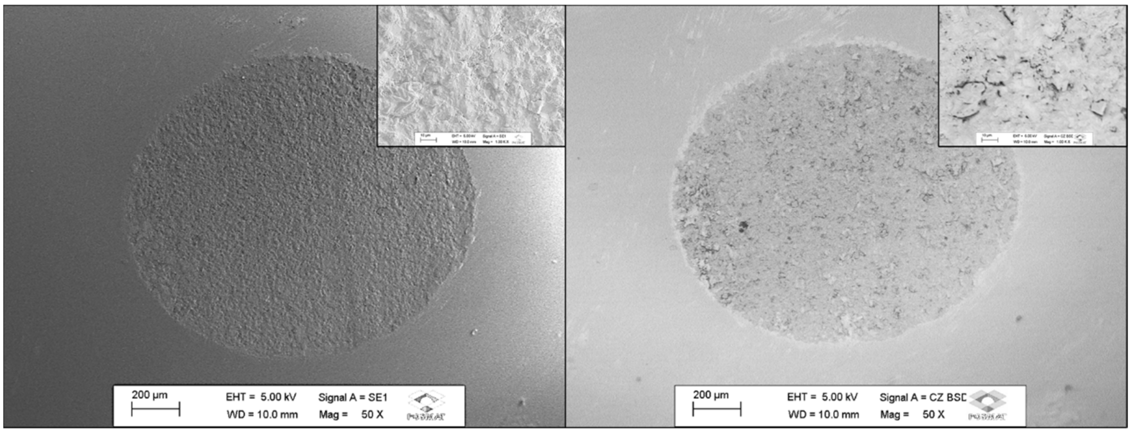

Fig. S1 – Representative SEM images of the worn surface of the alumina ball after the wear testing of the samples. Images were taken in the SE (left) and BSE (right) modes, with insets showing a zoomed view of the worn surface. The worn region was similar to all samples and confirms the degradation of the alumina ball during the wear tests.

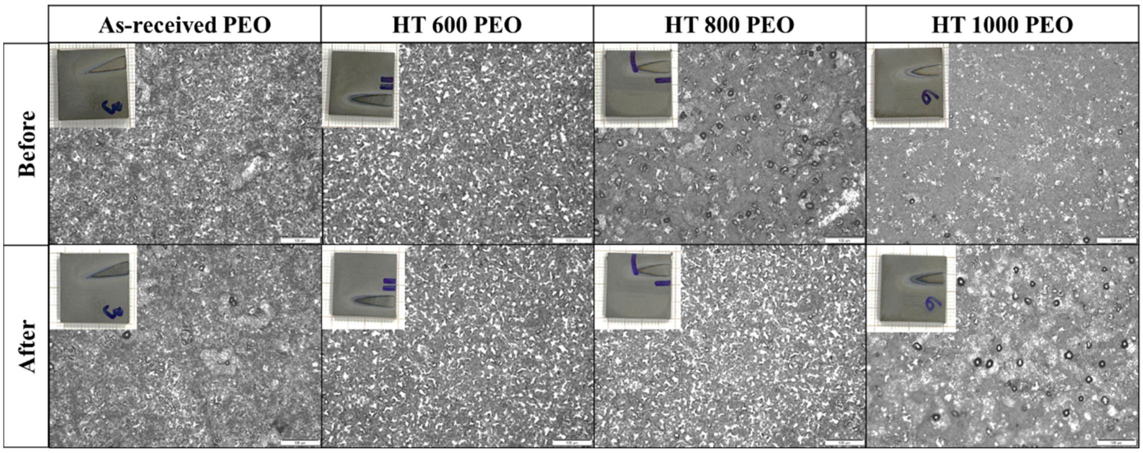

Fig. S2 – Topographical features of the samples before and after ultrasonic bath in distilled water for 30 min. The large images were obtained by SE-SEM, while the insets were acquired with a conventional camera. The similarity between the groups demonstrates the high adhesion of the coatings to the substrate.
